# Addressing Adherence Using Genotype-Specific PBPK Modeling—Impact of Drug Holidays on Tamoxifen and Endoxifen Plasma Levels

**DOI:** 10.3389/fphar.2017.00067

**Published:** 2017-03-14

**Authors:** Kristin J. R. Dickschen, Stefan Willmann, Georg Hempel, Michael Block

**Affiliations:** ^1^Computational Systems Biology, Bayer AGLeverkusen, Germany; ^2^Clinical Pharmacometrics, Bayer Pharma AGWuppertal, Germany; ^3^Clinical Pharmacy, Department of Pharmaceutical and Medical Chemistry, University of MuensterMuenster, Germany

**Keywords:** PBPK modeling, CYP2D6, genotype, drug holidays, endoxifen, tamoxifen, population simulation

## Abstract

**Introduction:** Tamoxifen is one of the most common treatment opportunities for hormonal positive breast cancer. Despite its good tolerability, patients demonstrate decreasing adherence over years impacting on therapeutic success. PBPK modeling was applied to demonstrate the impact of drug holidays on plasma levels of tamoxifen and its active metabolite endoxifen for different CYP2D6 genotypes.

**Materials and Methods:** A virtual study with 24,000 patients was conducted in order to investigate the development of tamoxifen steady-state kinetics in patient groups of different CYP2D6 genotypes. The impact of drug holidays on steady-state kinetics was investigated assuming changing drug holiday scenarios.

**Results:** Drug holidays in CYP2D6 extensive and intermediate metabolizers (EMs, IMs) exceeding 1 month lead to a decrease of endoxifen steady-state trough levels below the 5th percentile of the control group. Assuming drug holidays of 1, 2, or 3 months and administering a fixed-dose combination of 20 mg tamoxifen and 3 mg endoxifen EMs demonstrated re-established endoxifen steady-state trough levels after 5, 8, and 9 days. IMs receiving the same fixed-dose combination demonstrated re-established endoxifen steady-state trough levels after 7, 10, and 11 days.

**Discussion:** The PBPK model impressively demonstrates the impact of drug holidays in different CYP2D6 genotypes on PK. Population simulation results indicate that drug holidays of more than 2 weeks cause a tremendous decrease of plasma levels despite the long half-life of tamoxifen. To improve therapeutic success, PBPK modeling allows identifying genotype-specific differences in PK following drug holidays and adequate treatment with loading doses.

## Introduction

Tamoxifen treatment proved to be highly successful against hormone receptor positive breast cancer (Early Breast Cancer Trialists' Collaborative Group (EBCTCG), 2005). Recently, the American Society of Clinical Oncology (ASCO) updated its guidelines to recommend up to 10 years of adjuvant endocrine therapy. This adds an additional benefit on breast cancer survival for patients. Tamoxifen is characterized by excellent efficacy and good tolerability and treatment for up to 10 years is feasible (Burstein et al., 2014).

**Figure 1:**
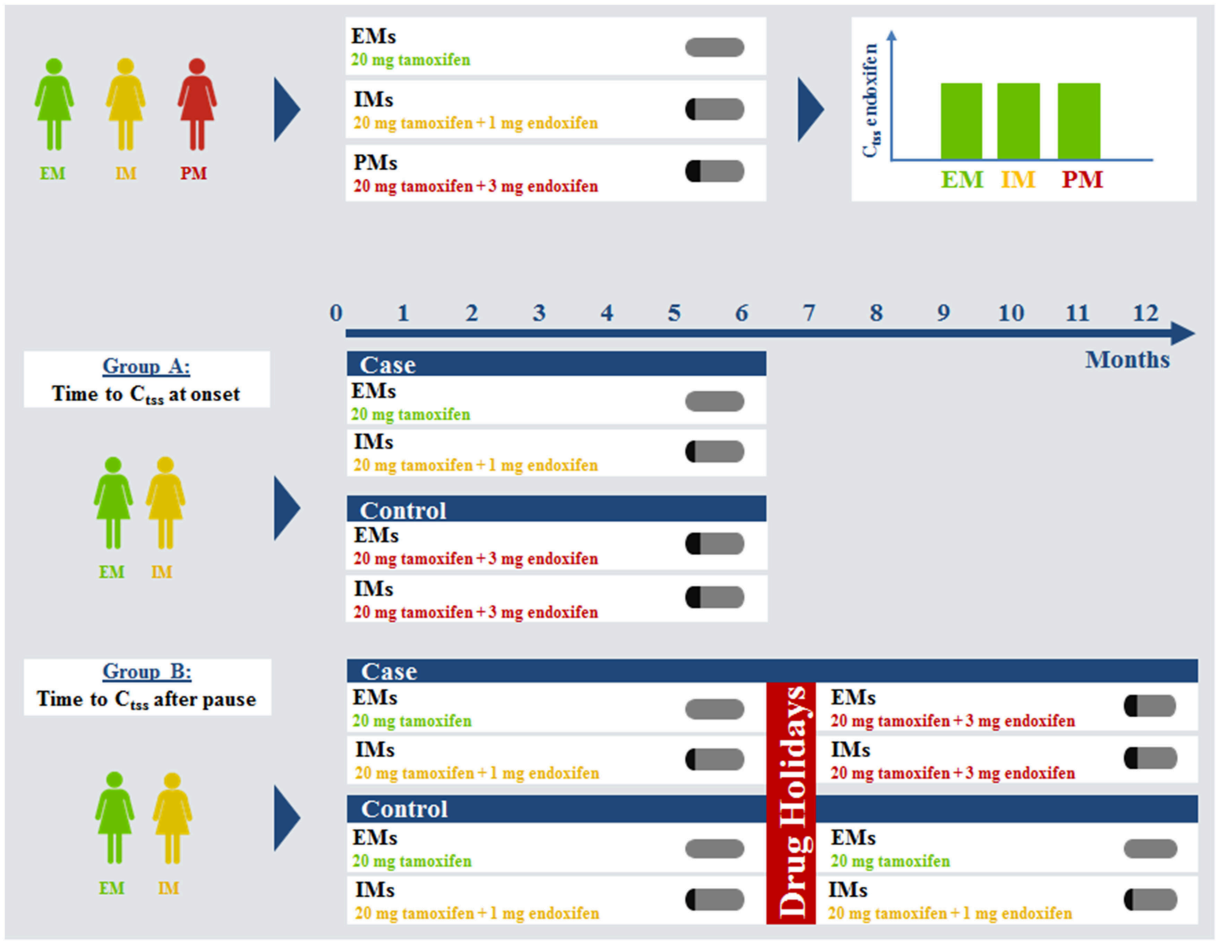
**Virtual clinical trial simulation rationale and study plan**. C_tss_, steady-state trough concentration; EM, extensive metabolizer; IM, intermediate metabolizer; PM, poor metabolizer.

It is acknowledged that the formation of tamoxifen's highly active metabolite endoxifen depends on the activity of the polymorphic enzyme cytochrome P450 2D6 (CYP2D6) (Brauch et al., 2008, 2009; Mürdter et al., 2011; Maximov et al., 2013). More than 100 alleles of CYP2D6 have already been reported (http://www.cypalleles.ki.se/cyp2d6.htm). Patients with the extensive metabolizer (EM) genotype show significantly higher endoxifen steady-state levels than patients with an intermediate metabolizer (IM) genotype than patients with a poor metabolizer (PM) genotype, each undergoing tamoxifen treatment with the standard dose of 20 mg daily (Schroth et al., 2007, 2009, 2010; Brauch et al., 2011, 2013a,b; Saladores et al., 2013; Schwab et al., 2013). In addition, the metabolic pattern in CYP2D6 EMs resulting out of standard tamoxifen is associated with better treatment outcome (Schroth et al., 2009, 2010). This led to the question how to improve tamoxifen treatment for patients, irrespective of their CYP2D6 genotype. Physiologically based pharmacokinetic (PBPK) modeling was used to address the issue of CYP2D6-dependent formation of endoxifen out of tamoxifen. PBPK-modeling separates substance-specific as well as anatomical and physiological species-specific parameters within a mechanistic framework of a generic PBPK model structure (for reviews please refer to Willmann et al., 2003; Nestorov, 2007). In the context of such a PBPK-model, the influence of CYP2D6 enzyme activity on endoxifen PK was already investigated. A fully coupled CYP2D6-genotype specific whole-body PBPK model of tamoxifen, N-desmethyl-tamoxifen, 4-hydroxy-tamoxifen, and endoxifen was established which provided valuable insight into the tamoxifen mass balance (Dickschen et al., 2012). The model was further used to assess, which dosing combinations are able to equalize endoxifen exposure in CYP2D6 IMs and PMs compared to EMs undergoing standard tamoxifen (20 mg/d) therapy. That approach resulted in suggested fixed-dose combinations of tamoxifen (20 mg/d) and CYP2D6 genotype-specific amounts of endoxifen for IMs (1 mg/d) and PMs (3 mg/d). These combinations will now be prospectively evaluated in a clinical intervention trial (Dickschen et al., 2014; Figure 1 upper row).

Along with the introduced differences for the therapeutic effect related to CYP2D6 genotype, patient adherence is a second key factor influencing treatment benefit. It was reported that patient adherence to tamoxifen progressively decreases over time, especially after 1 year of treatment (Partridge, 2006; Ruddy and Partridge, 2009; Ziller et al., 2009; Makubate et al., 2013). Thus, the change in adjuvant endocrine treatment paradigm from 5 to 10 years will probably face decreasing patient adherence as a problem. Decreased adherence is likely to occur more often in patients of the CYP2D6 EM genotype i.e., the patients that benefit most from standard tamoxifen treatment might have an increased risk of decreased efficacy (Rae et al., 2009). The fixed-dose combination of tamoxifen and endoxifen with or without therapeutic drug monitoring could prove useful in re-establishing steady-state kinetics following drug holidays in CYP2D6 EM and IM patients (Figure 1 Group B). Investigating the impact of drug holidays on tamoxifen PK in clinical daily practice for patients of different CYP2D6 genotypes is prone to erroneous conclusions. Patient adherence is difficult to assess and patients tend to rate their compliance better than it actually is. A prospective clinical trial investigating the impact of drug holidays on tamoxifen kinetics in breast cancer patients would expose patients with sub-therapeutic concentrations of tamoxifen/endoxifen. Healthy volunteers would be exposed to tamoxifen for an unacceptably long time (up to 12 months) due to its long half-life. Therefore, such studies are almost impossible to conduct due to ethical concerns.

Here, PBPK modeling provides a helpful tool to investigate various drug-holiday scenarios. In addition, development of steady-state kinetics under standard tamoxifen takes long, especially achieving effective concentrations of the secondary metabolite endoxifen. A fixed-dose combination of tamoxifen and endoxifen (20 and 3 mg/d, respectively) could prove useful in accelerating the establishment of the steady-state metabolic pattern, too (Figure 1 Group A).

The presented work shows how genotype-specific PBPK modeling can be used to address the relevant question of patient adherence undergoing long-term therapy and the subsequent impact on PK. Moreover, PBPK modeling opens the door for minimizing the risk of decreased treatment benefit due to lack of adherence when combined with therapeutic drug monitoring as it enables for the calculation of individualized doses to re-establish efficacious metabolic patterns in all patients, irrespective of their genotype.

Genotype-specific fixed dose combinations for IMs and PMs were shown to equalize endoxifen exposure at steady-state *in silico* (Dickschen et al., 2014) (upper row). The fixed-dose combination of 20 mg tamoxifen and 3 mg endoxifen following this rationale is regarded as a standard for PMs, thus they were no longer evaluated in this analysis. EMs and IMs are assumed to be more prone to decreased adherence and can benefit from this fixed-dose combination to accelerate time to endoxifen steady-state trough levels. Drug holidays were simulated for a time of 2, 4, 8, or 12 weeks.

## Materials and methods

The presented coupled PBPK model was developed by means of the computational systems biology software platform including PK-Sim® 4.2.4 and MoBi® 2.3.5 (www.systems-biology.com/products). PK-Sim® also calculated the PK-parameters from the simulation results. Population simulations were conducted using the MoBi® Toolbox for MATLAB® 2.2/2.3 (www.mathworks.com/products/matlab). This tool was also used for the evaluation of simulation results in terms of plasma concentration-time profiles, goodness-of-fit plots, box-whisker-plots, as well as calculation of median plasma concentrations of the simulated compounds.

A virtual study with two groups was conducted. In group A the time to development of endoxifen steady-state kinetics in CYP2D6 EMs under standard therapy (20 mg/d) and CYP2D6 IMs under fixed-dose combination (20 mg/d tamoxifen, 1 mg/d endoxifen) was investigated. Time to steady-state was the day before endoxifen C_tss_ of CYP2D6 EMs was exceeded (cases) or the day when endoxifen C_tss_ of CYP2D6 EMs was again achieved (controls). Results were compared to CYP2D6 EMs and IMs under the fixed-dose combination of 20 mg/d tamoxifen and 3 mg/d endoxifen. Thus, in group A, controls consisted of CYP2D6 EMs and IMs each receiving either standard tamoxifen (CYP2D6 EMs) or the CYP2D6 IM-specific fixed-dose combination once daily (CYP2D6 IMs) to ensure equalized endoxifen exposure in both genotypes. Cases consisted of CYP2D6 EMs and IMs receiving the CYP2D6 fixed-dose combination of 20 mg tamoxifen and 3 mg endoxifen once daily. Population simulations were conducted in each the four groups (*N* = 1000, respectively).

In group B the impact of drug holidays in CYP2D6 EMs and IMs after 6 months of tamoxifen was investigated. To ensure equalized endoxifen exposure in both genotype groups CYP2D6 EMs received standard tamoxifen and IMs received a fixed-dose combination (20 mg/d tamoxifen, 1 mg/d endoxifen). After 2, 4, 8, or 12 weeks of no drug intake administration of standard tamoxifen (EMs, controls) or 20 mg/d tamoxifen and 1 mg/d endoxifen (IMs, controls) were compared to the intake of 20 mg/d tamoxifen and 3 mg/d endoxifen in CYP2D6 EMs and IMs (cases). Thus, in group B, controls consisted of CYP2D6 EMs and IMs each receiving standard tamoxifen once daily (CYP2D6 EMs) or the CYP2D6 IM-specific fixed-dose combination once daily (CYP2D6 IMs) for 6 months. Subsequently, drug holidays of different lengths was simulated, namely for 2, 4, 8, and 12 weeks. Consequently, drug intake was re-established in terms of CYP2D6 EMs with standard tamoxifen once daily and in terms of CYP2D6 IMs with the CYP2D6 IM fixed-dose combination once daily until 12 months of intake were completed. Cases were made up of CYP2D6 EMs and IMs receiving standard tamoxifen once daily (CYP2D6 EMs) or the CYP2D6 IM-specific fixed-dose combination once daily (CYP2D6 IMs) for 6 months. Subsequently, drug holidays of different lengths was simulated, namely drug holidays taken for 2, 4, 8, and 12 weeks. Consequently, drug intake was re-established with CYP2D6 PM-specific fixed-dose combination in CYP2D6 EMs and IMs. Population simulations were conducted in each group (*N* = 1000, respectively).

The goal of the clinical trial simulation is to analyze the time to target concentration of END either at therapy onset or following drug holidays for CYP2D6 EMs and IMs. To match END exposure in both groups, IMs received their fixed dose combination of 20 mg tamoxifen and 1 mg endoxifen whereas EMs remained under standard therapy. Target concentration range in each study arm was the endoxifen steady-state trough concentration (C_tss_) observed in CYP2D6 EMs receiving standard tamoxifen assuming full adherence. Time to steady-state (either achievement or re-establishment) was the day before endoxifen C_tss_ of CYP2D6 EMs was exceeded (cases) or the day when endoxifen C_tss_ of CYP2D6 EMs was again achieved (controls). Overall, 20 different administration protocols in two study groups were investigated in 20,000 virtual female patients. The administration protocols investigated are illustrated in detail in Table 1.

**Table 1:** **Virtual clinical trial design for tamoxifen drug holidays and re-establishment of steady-state kinetics in CYP2D6 EMs and IMs**.

**Study Arm**	**Run-In Phase**	**Trial Phase**
**GROUP A**	**Month 1–Month 6**	**Month 7–Month 12**
**CYP2D6 EM**		
Case	20 mg TAM + 3.0 mg END q.d.	20 mg TAM + 3.0 mg END q.d.
Control	20 mg TAM q.d.	20 mg TAM q.d.
**CYP2D6 IM**		
Case	20 mg TAM + 3.0 mg END q.d.	20 mg TAM + 3.0 mg END q.d.
Control	20 mg TAM + 1.0 mg END q.d.	20 mg TAM + 1.0 mg END q.d.
**GROUP B**	**Month 1–Month 6**	**POST DRUG HOLIDAYS-MONTH 12**
**CYP2D6 EM**		
Case drug holidays 2 weeks	20 mg TAM q.d.	20 mg TAM + 3.0 mg END q.d.
Case drug holidays 4 weeks	20 mg TAM q.d.	20 mg TAM + 3.0 mg END q.d.
Case drug holidays 8 weeks	20 mg TAM q.d.	20 mg TAM + 3.0 mg END q.d.
Case drug holidays 12 weeks	20 mg TAM q.d.	20 mg TAM + 3.0 mg END q.d.
Control drug holidays 2 weeks	20 mg TAM q.d.	20 mg TAM q.d.
Control drug holidays 4 weeks	20 mg TAM q.d.	20 mg TAM q.d.
Control drug holidays 8 weeks	20 mg TAM q.d.	20 mg TAM q.d.
Control drug holidays 12 weeks	20 mg TAM q.d.	20 mg TAM q.d.
**CYP2D6 IM**		
Case drug holidays 2 weeks	20 mg TAM + 1.0 mg END q.d.	20 mg TAM + 3.0 mg END q.d.
Case drug holidays 4 weeks	20 mg TAM + 1.0 mg END q.d.	20 mg TAM + 3.0 mg END q.d.
Case drug holidays 8 weeks	20 mg TAM + 1.0 mg END q.d.	20 mg TAM + 3.0 mg END q.d.
Case drug holidays 12 weeks	20 mg TAM + 1.0 mg END q.d.	20 mg TAM + 3.0 mg END q.d.
Control drug holidays 2 weeks	20 mg TAM + 1.0 mg END q.d.	20 mg TAM + 1.0 mg END q.d.
Control drug holidays 4 weeks	20 mg TAM + 1.0 mg END q.d.	20 mg TAM + 1.0 mg END q.d.
Control drug holidays 8 weeks	20 mg TAM + 1.0 mg END q.d.	20 mg TAM + 1.0 mg END q.d.
Control drug holidays 12 weeks	20 mg TAM + 1.0 mg END q.d.	20 mg TAM + 1.0 mg END q.d.

From all simulations, median C_tss_ concentrations and percentiles were calculated. Target endoxifen C_tss_ is defined as the median C_tss_ of endoxifen in 1,000 CYP2D6 EMs receiving standard tamoxifen for 12 months assuming full adherence.

## Results

**Figure 2:**
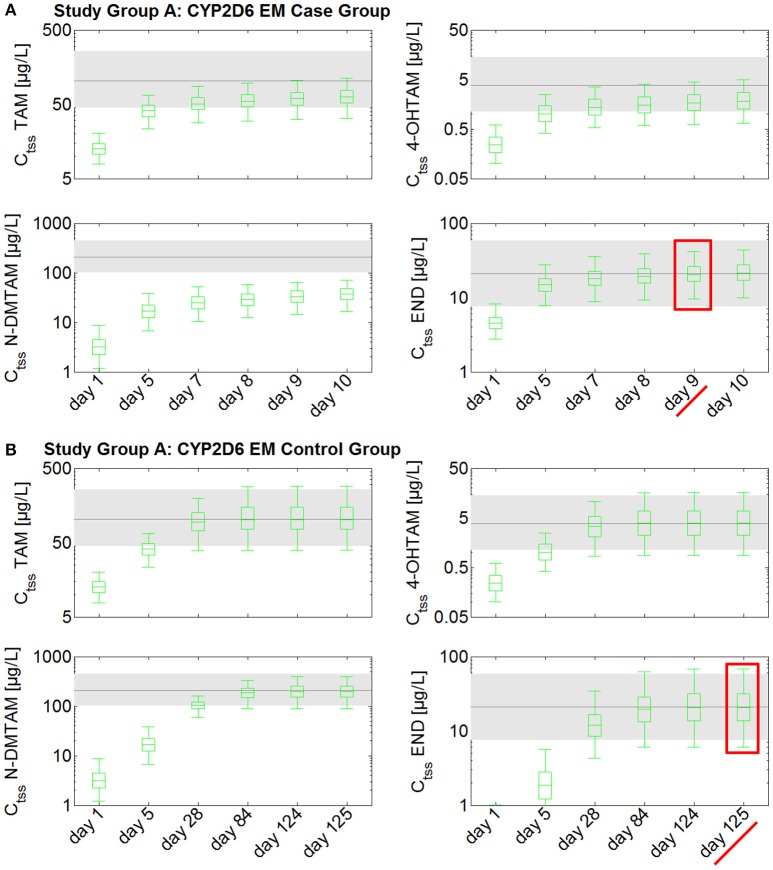
**Simulated time to steady-state in CYP2D6 EMs receiving either standard tamoxifen (control) or the fixed-dose combination (case)**. C_tss_ of TAM, NDM-TAM, 4-OHTAM, and END following a fixed-dose combination of 20 mg tamoxifen and 3 mg endoxifen in CYP2D6 EMs as a loading dose schedule (case) compared to a control group of CYP2D6 EMs receiving standard tamoxifen. Results are benchmarked to median C_tss_ levels of all four compounds in CYP2D6 EMs under standard tamoxifen (shaded areas, percentiles 5–95). **(A)** Simulation results in the case group receiving 20 mg tamoxifen and 3 mg endoxifen. **(B)** Simulation results in the control group receiving 20 mg tamoxifen. TAM, tamoxifen; N-DMTAM, N-desmethyltamoxifen; 4-OHTAM, 4-hydroxytamoxifen; END, endoxifen.

Steady-state kinetics of tamoxifen and its main metabolites following daily intake of 20 mg tamoxifen in CYP2D6 EMs take at least 4 months to develop (Figure 2). As endoxifen is a major contributor to tamoxifen's anti-tumoral activity, the established fixed-dose combination of 20 mg tamoxifen and 3 mg endoxifen could be used in order to reduce the time to endoxifen steady-state levels in CYP2D6 EMs. A comparison between the case group receiving 20 mg tamoxifen and 3 mg endoxifen, and the control group receiving 20 mg tamoxifen, shows that the case group reaches C_tss_ of endoxifen already after 9 days (Figure 2A). In the control group, however, C_tss_ of endoxifen is not reached before day 125 (Figure 2B). Thus, administration of the fixed-dose combination in CYP2D6 EMs starting tamoxifen treatment is able to considerably speed up the development of endoxifen C_tss_ by 116 days (Figures 2A,B).

**Figure 3:**
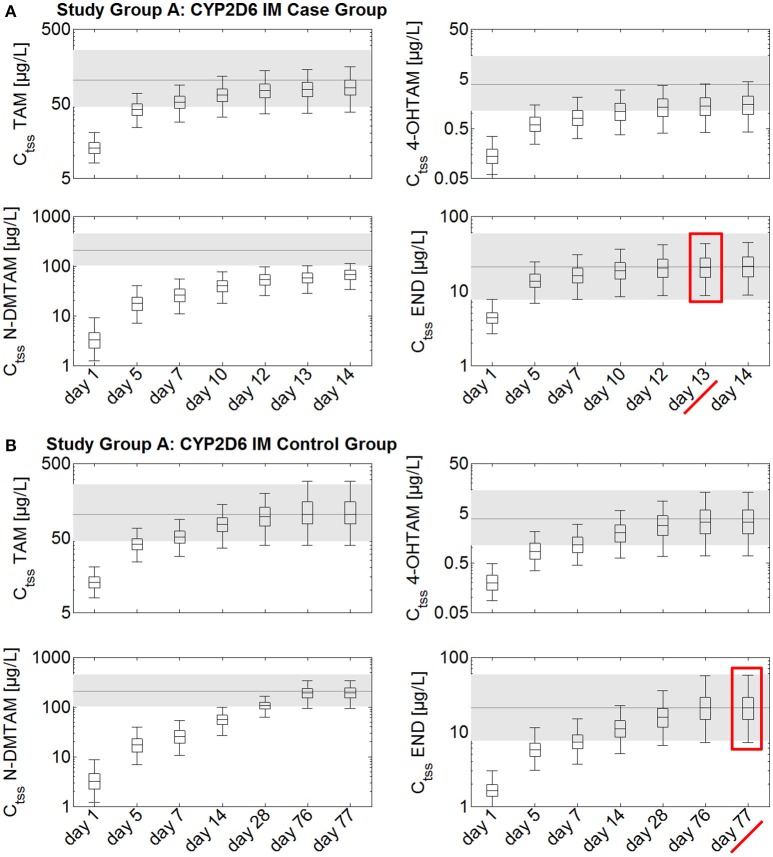
**Simulated time to steady-state in CYP2D6 IMs receiving either standard tamoxifen and 1 mg endoxifen (control) or standard tamoxifen and 3 mg endoxifen (case)**. C_tss_ of TAM, NDM-TAM, 4-OHTAM, and END following a fixed-dose combination of 20 mg tamoxifen and 3 mg endoxifen in CYP2D6 IMs as a loading dose schedule (case) compared to a control group of CYP2D6 IMs receiving standard tamoxifen and 1 mg endoxifen. Results are benchmarked to median C_tss_ levels of all four compounds in CYP2D6 EMs under standard tamoxifen (shaded areas, percentiles 5–95). **(A)** Simulation results in the case group receiving 20 mg tamoxifen and 3 mg endoxifen. **(B)** Simulation results in the control group receiving 20 mg tamoxifen and 1 mg endoxifen. TAM, tamoxifen; N-DMTAM, N-desmethyltamoxifen; 4-OHTAM, 4-hydroxytamoxifen; END, endoxifen.

Steady-state kinetics of tamoxifen, N-desmethyltamoxifen, 4-hydroxytamoxifen, and endoxifen following daily intake of 20 mg tamoxifen and 1 mg endoxifen in CYP2D6 IMs take at least 10–11 weeks to develop (Figure 3). A comparison between the case group receiving 20 mg tamoxifen and 3 mg endoxifen and the control group, receiving 20 mg tamoxifen and 1 mg endoxifen, shows that the case group reaches C_tss_ of endoxifen after 13 days (Figure 3A). In the control group, however, C_tss_ of endoxifen is not reached before day 77 (Figure 3B). The administration of the fixed-dose combination in CYP2D6 IMs starting tamoxifen treatment is able to considerably speed up the development of endoxifen C_tss_ by 54 days (Figures 3A,B).

Thus, CYP2D6 EMs receiving 20 mg tamoxifen and 3 mg endoxifen achieve endoxifen C_tss_ 4 days earlier than CYP2D6 IMs under the same treatment (Table 2).

**Table 2:** **Simulated time to endoxifen C_tss_ in CYP2D6 EMs and IMs under the treatment ensuring comparable exposure in both groups (controls) and the fixed-dose combination of 20 mg tamoxifen and 3 mg endoxifen (cases)**.

	**Time to endoxifen C_tss_ in CYP2D6 EMs [days]**	**Time to endoxifen C_tss_ in CYP2D6 IMs [days]**
**STUDY GROUP A**		
Case	9	13
Control	125	77
**STUDY GROUP B**		
Case drug holidays 2 weeks	2	4
Case drug holidays 4 weeks	5	7
Case drug holidays 8 weeks	8	10
Case drug holidays 12 weeks	9	11
Control drug holidays 2 weeks	126	40
Control drug holidays 4 weeks	>100	55
Control drug holidays 8 weeks	>100	65
Control drug holidays 12 weeks	>100	68

**Figure 4:**
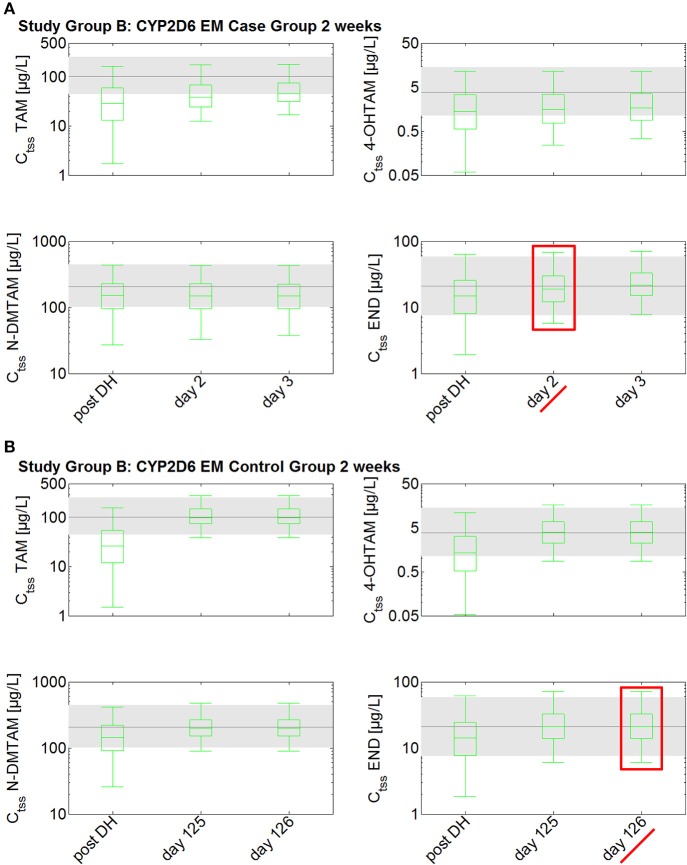
**Simulated time to steady-state in CYP2D6 EMs receiving either standard tamoxifen (control) or the fixed-dose combination (case) following drug holidays after 2 weeks**. C_tss_ of TAM, NDM-TAM, 4-OHTAM, and END following a fixed-dose combination of 20 mg tamoxifen and 3 mg endoxifen in CYP2D6 EMs as a loading dose schedule (case) after a 2-week drug holiday compared to a control group of CYP2D6 EMs receiving standard tamoxifen. Results are benchmarked to median C_tss_ levels of all four compounds in CYP2D6 EMs under standard tamoxifen (shaded areas, percentiles 5–95). **(A)** Simulation results in the case group receiving 20 mg tamoxifen and 3 mg endoxifen. **(B)** Simulation results in the control group receiving 20 mg tamoxifen. TAM, tamoxifen; N-DMTAM, N-desmethyltamoxifen; 4-OHTAM, 4-hydroxytamoxifen; END, endoxifen.

The investigation of the impact of drug holidays on tamoxifen PK was also simulated with the PBPK model. In the CYP2D6 EM population, drug holidays of already 2 weeks lead to a decrease of endoxifen trough concentration (C_t_) to a range around the 25th percentile of the median C_tss_ of endoxifen in CYP2D6 EMs under standard tamoxifen with full adherence (C_t_ after 2 weeks drug holidays: 14.9 μg/L vs. C_tss_ 25th percentile with full adherence: 13.8 μg/L) (Figure 4). Drug holidays exceeding 1 month lead to a decrease of endoxifen C_t_ under the 5th percentile of endoxifen C_tss_ in CYP2D6 EMs under standard tamoxifen assuming full adherence. To accelerate the re-establishment of endoxifen C_tss_ in CYP2D6 EMs taking drug holidays of 2 weeks, 1, 2, or 3 months, the fixed-dose combination of 20 mg tamoxifen and 3 mg endoxifen was compared to the standard tamoxifen dose. Patients with drug holidays of 2 weeks followed by the intake of the fixed-dose combination of 20 mg tamoxifen and 3 mg endoxifen show a re-establishment of endoxifen C_tss_ already after 2 days whereas patients receiving standard tamoxifen after 2 weeks of drug holidays only show a re-establishment of endoxifen C_tss_ at day 307 which is day 126 post first-dose of re-starting tamoxifen intake on day 182.

The situation pronouncedly worsened when drug holidays of 1, 2, or 3 months were simulated (data not shown, Table 2). Patients receiving the fixed-dose combination of 20 mg tamoxifen and 3 mg endoxifen demonstrate re-established endoxifen C_tss_ after 5, 8, and 9 days of taking the fixed-dose combination. However, patients taking only standard tamoxifen after the drug holidays of 1, 2, or 3 months length, do not exert endoxifen C_tss_ before day 336 which was end of simulation time. Thus, re-establishment of endoxifen C_tss_ only by taking standard tamoxifen following drug holidays takes at least 100 days following 1 month of no tamoxifen intake and probably worsened with increasing length of no drug intake.

**Figure 5:**
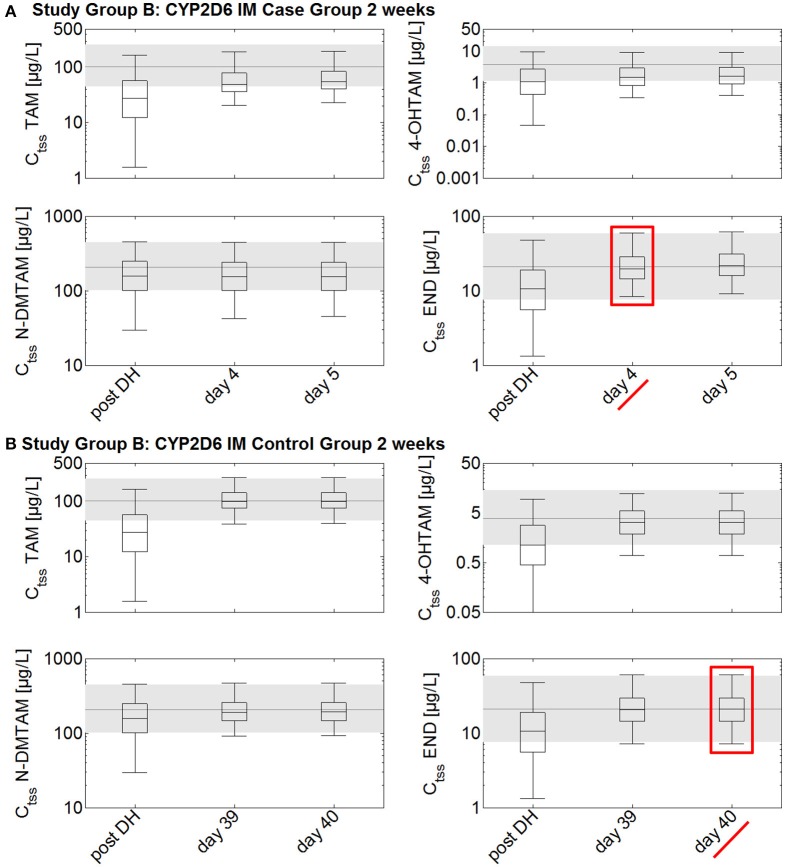
**Simulated time to steady-state in CYP2D6 IMs receiving either standard tamoxifen and 1 mg endoxifen (control) or the fixed-dose combination of standard tamoxifen and 3 mg endoxifen (case) following drug holidays after 2 weeks**. C_tss_ of TAM, NDM-TAM, 4-OHTAM, and END following a fixed-dose combination of 20 mg tamoxifen and 3 mg endoxifen in CYP2D6 IMs as a loading dose schedule (case) after a 2-week drug holiday compared to a control group of CYP2D6 IMs receiving standard tamoxifen and 1 mg endoxifen. Results are benchmarked to median C_tss_ levels of all four compounds in CYP2D6 EMs under standard tamoxifen (shaded areas, percentiles 5–95). **(A)** Simulation results in the case group receiving 20 mg tamoxifen and 3 mg endoxifen. **(B)** Simulation results in the control group receiving 20 mg tamoxifen and 1 mg endoxifen. TAM, tamoxifen; N-DMTAM, N-desmethyltamoxifen; 4-OHTAM, 4-hydroxytamoxifen; END, endoxifen.

In the CYP2D6 IM population, drug holidays of 2 weeks lead to a decrease of endoxifen C_t_ to a range lower than the 25th percentile of the median C_tss_ of endoxifen in CYP2D6 EMs under standard tamoxifen with full adherence (C_t_ after 2 weeks drug holidays: 10.6 μg/L vs. C_tss_ 25th percentile with full adherence: 13.8 μg/L; Figure 5). Drug holidays exceeding 1 month lead to a decrease of endoxifen C_t_ under the 5th percentile of endoxifen C_tss_ in CYP2D6 EMs under standard tamoxifen with full adherence.

To accelerate the re-establishment of endoxifen C_tss_ in CYP2D6 IMs taking drug holidays of 2 weeks, 1, 2, or 3 months, the fixed-dose combination of 20 mg tamoxifen and 3 mg endoxifen was compared to the fixed-dose combination of 20 mg tamoxifen and 1 mg endoxifen. Patients taking drug holidays of 2 weeks followed by the intake of the fixed-dose combination of 20 mg tamoxifen and 3 mg endoxifen show a re-establishment of endoxifen C_tss_ already after 4 days. Patients taking drug holidays of 2 weeks followed by the intake of 20 mg tamoxifen and 1 mg endoxifen only show a re-establishment of endoxifen C_tss_ at day 221 which is day 40 post first-dose of re-starting tamoxifen intake on day 182.

The situation again pronouncedly worsened when drug holidays of 1, 2, or 3 months were simulated. Patients receiving the fixed-dose combination of 20 mg tamoxifen and 3 mg endoxifen demonstrate re-established endoxifen C_tss_ after 7, 10, and 11 days of taking the fixed-dose combination. Patients taking only 20 mg tamoxifen and 1 mg endoxifen after the drug holidays of 1, 2, or 3 months length only show endoxifen C_tss_ after 55, 65, and 68 days.

Thus, CYP2D6 EMs receiving 20 mg tamoxifen and 3 mg endoxifen achieve endoxifen C_tss_ 2 days earlier than CYP2D6 IMs under the same treatment, irrespective of the length of drug holidays taken and simulated (Table 2). Moreover, time to steady-state re-establishment in CYP2D6 EMs following standard tamoxifen therapy takes longer than simulated, exposing patients to sub-therapeutic endoxifen C_tss_ for at least 3–4 months following drug holidays of more than 4 weeks. This gives a comparable picture when starting tamoxifen treatment in CYP2D6 EMs under standard tamoxifen treatment. CYP2D6 IMs receiving the fixed-dose combination of 20 mg tamoxifen and 1 mg endoxifen, leading to comparable endoxifen C_tss_ to CYP2D6 EMs under 20 mg tamoxifen need 6–9 weeks to re-establish endoxifen benchmark C_tss_.

## Discussion

For more than 40 years tamoxifen is now a cornerstone in the armamentarium against early hormone receptor positive breast cancer. Recently, adjuvant endocrine therapy paradigm has been adapted and ASCO now recommends treating women for up to 10 years, either within a sequential therapy of tamoxifen and aromatase inhibitors or even with tamoxifen alone. Tamoxifen is known for its excellent efficacy and good tolerability, thus treating patients for 10 years is feasible (Burstein et al., 2014). However, patient adherence to long term therapy is known to decrease over time and might represent a problem for treatment efficacy (Partridge, 2006; McCowan et al., 2008; Ruddy and Partridge, 2009; Ziller et al., 2009; Dezentje et al., 2010; Hershman et al., 2010; Makubate et al., 2013). As tamoxifen metabolism is prone to CYP2D6 activity, on top of the question how drug holidays impact on tamoxifen PK in general, this is of special interest for the different CYP2D6 genotypes in order to ensure full efficacy for all patients receiving tamoxifen (Stearns et al., 2003; Rae et al., 2009). The characteristic metabolite pattern resulting from tamoxifen daily dosing of 20 mg to CYP2D6 EMs was demonstrated to be well tolerated and highly beneficial over the past four decades (Early Breast Cancer Trialists' Collaborative Group (EBCTCG), 1992, 1998, 2005; Stearns et al., 2003, 2004). However, steady-state kinetics of tamoxifen metabolites require a very long period of time to develop which can be in the range of 3–4 months as demonstrated by our previously developed PBPK model (Wu et al., 2009; Dickschen et al., 2012, 2014). It was reported that endoxifen is the key contributor to tamoxifen's anti-tumoral activity exerting a concentration-dependent effect on tumor proliferation and growth (Johnson et al., 2004; Lim et al., 2005, 2006; Wu et al., 2009). Thus, the time to endoxifen C_tss_ is likely to be relevant for the full therapeutic benefit of tamoxifen. As it is well-described in literature that endoxifen formation out of tamoxifen depends on CYP2D6 activity, time to endoxifen C_tss_ and the level itself depend to a great extent on the CYP2D6 genotype (Stearns et al., 2003; Borges et al., 2006; Antunes et al., 2012; Damodaran et al., 2012; Maximov et al., 2013; Saladores et al., 2013).

The fixed-dose combination of 3 mg endoxifen and 20 mg tamoxifen, originally intended to optimize treatment in CYP2D6 PMs by equalizing endoxifen steady-state exposure compared to CYP2D6 EMs under standard tamoxifen, was used in simulations to investigate the impact on time to endoxifen steady-state levels in CYP2D6 EMs and IMs (Dickschen et al., 2012, 2014). In the presented virtual clinical trial, population simulation results demonstrate impressively that endoxifen steady-state levels develop far more quickly when the fixed-dose combination of 3 mg endoxifen and 20 mg tamoxifen was given to CYP2D6 EMs and IMs instead of standard tamoxifen to CYP2D6 EMs (116 days more quickly) or 1 mg endoxifen and 20 mg tamoxifen to CYP2D6 IMs (54 days more quickly). It was chosen to administer the previously published fixed-dose combination of 1 mg endoxifen and 20 mg tamoxifen daily as a control dose to the CYP2D6 IMs in this virtual clinical trial in order to achieve comparable endoxifen C_tss_ for all CYP2D6 genotypes (Dickschen et al., 2014). The presented results nicely outline that CYP2D6 EMs and IMs can achieve the effective endoxifen exposure faster when they start tamoxifen treatment with the fixed-dose combination of 20 mg tamoxifen and 3 mg endoxifen. Here, a time of 10 days would be sufficient for CYP2D6 EMs based on simulation results and 2 weeks for CYP2D6 IMs. After this loading dose schedule, the optimal tamoxifen treatment schedule would be 20 mg tamoxifen for CYP2D6 EMs and 1 mg endoxifen and 20 mg tamoxifen for CYP2D6 IMs according to previously published simulation results. Following this published individualized tamoxifen treatment rationale, CYP2D6 PMs would receive 20 mg tamoxifen and 3 mg endoxifen as a standard regimen. Thus, they were not longer part of this specific analysis. Another big concern in adjuvant endocrine long-term therapy is patient adherence and persistence to treatment (Partridge, 2006; Barron et al., 2007; McCowan et al., 2008; Rae et al., 2009; Ruddy and Partridge, 2009; Ziller et al., 2009; Dezentje et al., 2010; Hershman et al., 2010; Makubate et al., 2013). This is by now of special importance for adjuvant endocrine treatment as tamoxifen treatment duration is likely to increase from 5 to 10 years and patient adherence is known to decrease over time (Hershman et al., 2010). Moreover, it was reported that especially in CYP2D6 EMs discontinuation of tamoxifen treatment is more likely than in patients with impaired CYP2D6 enzyme activity (Rae et al., 2009). A potential reason could be an increased rate of adverse events such as hot flushed in CYP2D6 EMs. Thus, paradoxically adherence in patients most likely to benefit to a far greater extent from standard tamoxifen treatment is worse than in patients less likely to receive full benefit of regular tamoxifen treatment, i.e., with an impaired CYP2D6 enzyme activity. However, it is very difficult to estimate patient adherence in investigational clinical trials as self-reported adherence pronouncedly deviates from true patient adherence (Ziller et al., 2009). Here, the developed and qualified PBPK model provided a very useful tool to investigate the impact of drug holidays in CYP2D6 EMs and IMs on plasma levels. Population simulation results in 20,000 virtual subjects indicate that drug holidays of more than 2 weeks cause a tremendous decrease of plasma levels of all four substances investigated despite the long half-life of tamoxifen (Fuchs et al., 1996). Steady-state levels of endoxifen are not re-established before 100 days of intake of 20 mg tamoxifen in the CYP2D6 EM groups following drug holidays of 2 weeks or more. In the CYP2D6 IM control group, patients show re-established endoxifen steady-state levels after 40, 55, 65, or 68 days following drug holidays of 2 weeks, 1, 2, or 3 months and subsequent intake of 1 mg endoxifen and 20 mg tamoxifen, respectively. Thus, CYP2D6 EMs are rendered with subtherapeutic endoxifen levels for at least 3–4 months following drug holidays of 4 weeks and more and CYP2D6 IMs for 6 weeks or more assuming that they already receive an fixed-dose combination of 1 mg endoxifen and 20 mg tamoxifen to equalize their endoxifen C_tss_ to the one observed in CYP2D6 EMs under standard tamoxifen. In both case groups, CYP2D6 EMs and CYP2D6 IMs, the intake of 3 mg endoxifen and 20 mg tamoxifen significantly speeds up the re-establishment of endoxifen steady-state levels. CYP2D6 EMs show endoxifen steady-state levels after 2, 5, 8, or 9 days following 2 weeks, 1, 2, or 3 of drug holidays and subsequent intake of 3 mg endoxifen and 20 mg tamoxifen, respectively. CYP2D6 IMs show endoxifen steady-state levels after 4, 7, 10, or 11 days following 2 weeks, 1, 2, or 3 months of drug holidays and subsequent intake of 3 mg endoxifen and 20 mg tamoxifen. Consequently, the time of subtherapeutic endoxifen C_tss_ in both genotype groups is reduced to <2 weeks even when patients stop intake for 3 months which is usually the time frame between two prescriptions. Due to the decreased endogenous endoxifen formation out of the standard dose of tamoxifen, CYP2D6 IMs take in general 2 days longer to re-establish endoxifen C_tss_ when receiving exactly the same fixed dose combination as CYP2D6 EMs. This demonstrates the impact of the CYP2D6 genotype on endoxifen formation once more and is well in line with current knowledge about CYP2D6 activity as a PK-relevant prognostic factor in tamoxifen treatment (Brauch et al., 2013a).

The fixed-dose combination of 3 mg endoxifen and 20 mg tamoxifen is thus not only useful for optimizing tamoxifen treatment in CYP2D6 PMs as proposed previously but can be also very useful in optimizing tamoxifen treatment of CYP2D6 EMs and IMs with decreased adherence (Rae et al., 2009; Dickschen et al., 2014). The virtual clinical trial impressively demonstrates the differing impact of drug holidays on PK in patients with distinct CYP2D6 genotypes. The application of the fixed-dose combination of tamoxifen and endoxifen in CYP2D6 EMs and IMs however might be supported by therapeutic drug monitoring of patients to determine plasma concentration levels of tamoxifen and its metabolites as self-reported patient adherence is most likely not representative for true adherence and plasma concentration levels of tamoxifen and endoxifen should determine the use and duration of the proposed fixed-dose combination. Overall, this investigation demonstrates the value of PBPK simulations in situations where clinical studies are almost impossible to conduct taking into account the impact of different genotypes on PK. The simulations provide guidance for clinical investigations with the opportunity to optimize endocrine therapy with the potential to improve clinical outcome in breast cancer patients.

## Author contributions

KD performed the analysis including the model development and all simulations, and wrote the manuscript. SW and GH contributed to the concept development and evaluation, and was involved in writing and reviewing the manuscript. MB contributed to the analysis and interpretation of modeling results, writing, and reviewing the manuscript.

## Funding

MB and KD acknowledge financial support by the Project “TAMENDOX: Verbesserte endokrine Therapie beim Mamakarzinom” (01EK1509C) funded by the German Federal Ministry of Education and Research (BMBF). This work was financially supported by a research grant from Bayer Technology Services GmbH as part of a Ph.D. thesis of KD. The research grant was received by GH (between 2010 and 2013).

### Conflict of interest statement

KD and MB are employed by Bayer AG. SW is employed by Bayer Pharma AG. A patent for fixed-dose combinations of tamoxifen and endoxifen has been filed by KD and SW. GH received research grants from Bayer Technology Services GmbH, now Bayer AG that the research was conducted in the absence of any commercial or financial relationships that could be construed as a potential conflict of interest.
